# Supplementary material for: Pyruvate Dehydrogenase Kinase 1 inhibition mediated oxidative phosphorylation enhancement in cartilage promotes osteoarthritis progression
Source: BMC Musculoskelet Disord. 2023 Jul 20;24:597. doi: 10.1186/s12891-023-06585-6 (PMC10357736; doi:10.1186/s12891-023-06585-6)
Supplement: Supplementary file 1 — Additional file 1. [file 12891_2023_6585_MOESM1_ESM.zip › BP.Gsea.1653623667859/GOBP_REGULATION_OF_NUCLEAR_TRANSCRIBED_MRNA_CATABOLIC_PROCESS_DEADENYLATION_DEPENDENT_DECAY.html]

Details for gene set GOBP\_REGULATION\_OF\_NUCLEAR\_TRANSCRIBED\_MRNA\_CATABOLIC\_PROCESS\_DEADENYLATION\_DEPENDENT\_DECAY[GSEA]

|  || Dataset | 55457\_collapsed\_to\_symbols.cls.cls #OA\_versus\_normal.cls.cls #OA\_versus\_normal\_repos |
| Phenotype | cls.cls#OA\_versus\_normal\_repos |
| Upregulated in class | normal |
| GeneSet | GOBP\_REGULATION\_OF\_NUCLEAR\_TRANSCRIBED\_MRNA\_CATABOLIC\_PROCESS\_DEADENYLATION\_DEPENDENT\_DECAY |
| Enrichment Score (ES) | -0.6097835 |
| Normalized Enrichment Score (NES) | -1.8022178 |
| Nominal p-value | 0.0034246575 |
| FDR q-value | 0.09659267 |
| FWER p-Value | 0.972 |
Table: GSEA Results Summary

  

Fig 1: Enrichment plot: GOBP\_REGULATION\_OF\_NUCLEAR\_TRANSCRIBED\_MRNA\_CATABOLIC\_PROCESS\_DEADENYLATION\_DEPENDENT\_DECAY      
 Profile of the Running ES Score & Positions of GeneSet Members on the Rank Ordered List

  

| SYMBOL | TITLE | RANK IN GENE LIST | RANK METRIC SCORE | RUNNING ES | CORE ENRICHMENT || 1 | CNOT7 | CCR4-NOT transcription complex subunit 7 [Source:HGNC Symbol;Acc:HGNC:14101] | 1676 | 0.402 | -0.0777 | No |
| 2 | CNOT1 | CCR4-NOT transcription complex subunit 1 [Source:HGNC Symbol;Acc:HGNC:7877] | 3842 | 0.172 | -0.2234 | No |
| 3 | HNRNPD | heterogeneous nuclear ribonucleoprotein D [Source:HGNC Symbol;Acc:HGNC:5036] | 4830 | 0.085 | -0.2889 | No |
| 4 | PAIP1 | poly(A) binding protein interacting protein 1 [Source:HGNC Symbol;Acc:HGNC:16945] | 4851 | 0.083 | -0.2796 | No |
| 5 | TOB1 | "transducer of ERBB2, 1 [Source:HGNC Symbol;Acc:HGNC:11979]" | 7892 | -0.147 | -0.4964 | No |
| 6 | CPEB3 | cytoplasmic polyadenylation element binding protein 3 [Source:HGNC Symbol;Acc:HGNC:21746] | 8060 | -0.160 | -0.4885 | No |
| 7 | YBX1 | Y-box binding protein 1 [Source:HGNC Symbol;Acc:HGNC:8014] | 9623 | -0.285 | -0.5727 | Yes |
| 8 | HNRNPU | heterogeneous nuclear ribonucleoprotein U [Source:HGNC Symbol;Acc:HGNC:5048] | 9847 | -0.306 | -0.5501 | Yes |
| 9 | TENT4A | terminal nucleotidyltransferase 4A [Source:HGNC Symbol;Acc:HGNC:16705] | 10003 | -0.322 | -0.5203 | Yes |
| 10 | ZFP36L2 | ZFP36 ring finger protein like 2 [Source:HGNC Symbol;Acc:HGNC:1108] | 10509 | -0.374 | -0.5107 | Yes |
| 11 | CSDE1 | cold shock domain containing E1 [Source:HGNC Symbol;Acc:HGNC:29905] | 10559 | -0.379 | -0.4650 | Yes |
| 12 | DHX9 | DExH-box helicase 9 [Source:HGNC Symbol;Acc:HGNC:2750] | 10627 | -0.386 | -0.4200 | Yes |
| 13 | SYNCRIP | synaptotagmin binding cytoplasmic RNA interacting protein [Source:HGNC Symbol;Acc:HGNC:16918] | 11182 | -0.454 | -0.4038 | Yes |
| 14 | AGO2 | argonaute RISC catalytic component 2 [Source:HGNC Symbol;Acc:HGNC:3263] | 11246 | -0.463 | -0.3483 | Yes |
| 15 | TNRC6B | trinucleotide repeat containing adaptor 6B [Source:HGNC Symbol;Acc:HGNC:29190] | 11947 | -0.582 | -0.3268 | Yes |
| 16 | PABPC1 | poly(A) binding protein cytoplasmic 1 [Source:HGNC Symbol;Acc:HGNC:8554] | 12562 | -0.778 | -0.2731 | Yes |
| 17 | ZFP36 | ZFP36 ring finger protein [Source:HGNC Symbol;Acc:HGNC:12862] | 12846 | -1.052 | -0.1580 | Yes |
| 18 | ZFP36L1 | ZFP36 ring finger protein like 1 [Source:HGNC Symbol;Acc:HGNC:1107] | 12888 | -1.244 | 0.0009 | Yes |
Table: GSEA details [plain text format]

  

Fig 2: GOBP\_REGULATION\_OF\_NUCLEAR\_TRANSCRIBED\_MRNA\_CATABOLIC\_PROCESS\_DEADENYLATION\_DEPENDENT\_DECAY      
 Blue-Pink O' Gram in the Space of the Analyzed GeneSet

  

Fig 3: GOBP\_REGULATION\_OF\_NUCLEAR\_TRANSCRIBED\_MRNA\_CATABOLIC\_PROCESS\_DEADENYLATION\_DEPENDENT\_DECAY: Random ES distribution      
 Gene set null distribution of ES for **GOBP\_REGULATION\_OF\_NUCLEAR\_TRANSCRIBED\_MRNA\_CATABOLIC\_PROCESS\_DEADENYLATION\_DEPENDENT\_DECAY**
